# The Burden of Disability in Africa and Cameroon: A Call for Optimizing the Education in Physical and Rehabilitation Medicine

**DOI:** 10.3389/fresc.2022.873362

**Published:** 2022-05-19

**Authors:** Giuseppe Cannata, Maurice Douryang, Concetta Ljoka, Laura Giordani, Marco Monticone, Calogero Foti

**Affiliations:** ^1^Physical Medicine and Rehabilitation, Clinical Sciences and Translational Medicine Department, Tor Vergata University, Rome, Italy; ^2^Department of Physiotherapy and Physical Medicine, University of Dschang, Dschang, Cameroon; ^3^Physical Medicine and Rehabilitation Unit, Tor Vergata Hospital, Rome, Italy; ^4^Physical Medicine and Rehabilitation, Department of Medical Sciences and Public Health, University of Cagliari, Cagliari, Italy

**Keywords:** training, physiotherapy, reeducation, impairments, handicap, health system, low- and middle-income countries, review

## Introduction

Physical and Rehabilitation Medicine (PRM) is the medical specialty of body functioning. Its aim is to diagnose, prevent and reduce disability resulting from the interaction between people and their environment (1, 2).

The World Health Organization (WHO) Global Disability Action Plan 2014–2021 “Better health for all people with disabilities” is a significant step toward rehabilitation services. For a successful implementation of the WHO recommendations, PRM organizations and individual PRM specialists are urged to contribute to the improvement of PRM services worldwide (3).

The International Classification of Functioning, Disability and Health (ICF) provides a widely accepted conceptual model and taxonomy of human functioning (1).

PRM specialists guarantee the citizen's access to rehabilitation services as a human right (4–6).

It is well-known that rehabilitation is essential to lower healthcare costs by decreasing the number of days spent in hospital, and that reducing disability improves quality of life. Varela et al. highlight the need for more scientific studies on the benefits of rehabilitation even in the preoperative phase, while we know that current studies confirm that early postoperative rehabilitation decreases pain and its consequences (7).

In long-term disabilities, the rehabilitation process for patients with complex problems requires a carefully planned and integrated program by the PRM physician who provides advice on diagnosis, prognosis, treatment options and risks for the patient and family. The PRM physician must take his leadership role over the rehabilitation team, as well as assist in the development of treatment protocols; his holistic perspective on long-term rehabilitation management makes a unique contribution (8).

High-quality rehabilitation care is a constituent element of healthcare systems worldwide. The implementation of these standards needs to be validated worldwide with particular attention to low- and middle-income countries. The standardization of staff training at both undergraduate and postgraduate levels is a key element in ensuring the highest standard of rehabilitation care. International bodies, such as the UEMS Board for PRM or the International Society for PRM, have already delivered normative documents setting standards in postgraduate PRM training.

They highlight the need to develop competency-based education, training physicians with the proper skills and knowledge required to meet the healthcare needs of people with disabilities, as a powerful mechanism to align education and training with health system priorities.

This is of particular value for countries with limited resources, where the knowledge and skills of rehabilitation physicians need to reflect not only the health profile of the population, but also the strengths and weaknesses of the health system.

## The Burden of Disability in Africa and Cameroon

As life expectancy increases, disability rates caused by the diseases listed above increase (9, 10).

The World Disability Report published by WHO in 2011 also states that the prevalence of severe and moderate disability is higher in Africa than in many other regions of the world especially in younger (<60 years) population groups (11). It is assumed that the causes are related to infectious diseases and injuries although the literature has limited evidence (12, 13).

A number of publications report a prevalence of disability in the general population ranging from 1.7% in Mali (14) to 17.1% in Sierra Leone (15), but it should be noted that these studies have used different methodologies and tools.

Disability prevalence in Cameroon was recently estimated in a survey of a sample of 1,617 adults aged 18+ using the Washington Group tools, which capture self-reported activity limitations in functional domains described in the ICF (16). There are several Washington Group modules recommended for adult populations: Short Set (6 items focusing on a subset of “core” functional domains such as seeing, hearing, mobility, memory/concentration, self-care, and communication); Labor Force Survey Disability Module (additional domains of anxiety and depression, 10 items); Short Set Enhanced (additional domain of upper body strength, 12 items); Extended Set on Functioning (additional domains of pain and fatigue, 17 items). The standard pre-determined threshold recommended for calculating internationally comparable disability prevalence data is to include anyone reporting a lot of difficulty or inability to do in any domain, and a wider threshold (some difficulty or worse) is often reported too. The prevalence of disability in this population tended to increase as modules were included with an additional number of items and using a wider threshold of functional limitations. Based on the Short Set, it ranged from 6.1% using the standard threshold to 66.3% using the wide threshold; based on the full Extended Set on Functioning, it ranged from 12.9% using the standard threshold to 71.0% using the wide threshold.

A study in a health district in Cameroon showed that many disabilities, such as orthopedic problems (mainly fractures), infectious diseases and neurological disabilities (mainly hemiplegia, hemiparesis and monoplegia), were due to traffic accidents and inappropriate medical interventions (17). In Mali congenital abnormalities, trauma, polio and leprosy were reported to be the most common causes (14), while in Liberia mental health disabilities were related to war and postwar experiences (18).

A large number of studies explored the effect of disability on health, education, social participation and livelihoods of people with disabilities.

Adults with disabilities were more likely to experience serious health problems and report limited access to healthcare and rehabilitation services (19).

A literature review published in 2018 on five West African countries defined important policy and program implications as follows:

(1) Application of standardized tools for monitoring the implementation of programs and policies at national level;
(2) Improving stakeholder coordination mechanisms at the country level;
(3) Supporting countries in using unified approaches to measuring disability and social exclusion;
(4) Strengthening the rigor of the evaluations of the effectiveness of disability-specific interventions;
(5) Disaggregation of routine data from development programs by disability (10).

A disability research team established the need to define strategies to improve the activities of daily living of people with disabilities in Cameroon (20).

A descriptive cross-sectional study pointed out that disabled people, and children in particular, are still marginalized, vulnerable and with little chance of recovery. Therefore, there is a clear need to improve the quality and availability of rehabilitative care with programmatic interventions that improve the accessibility to rehabilitation services for people with disabilities, provide them with the necessary safeguards, ensure implementation of existing laws, and neutralize any barrier to their social participation (21).

Regarding disability associated with human immunodeficiency virus (HIV) infection, a 2019 study shows that antiretroviral therapy improves impaired immune function. It is reported in the literature that physical (aerobic/endurance) exercises also seem to induce beneficial effects (22).

In another 2019 study, Ibeneme et al. argue that while aerobic exercise does not improve levels of inflammatory biomarkers (IL-6 and IL-1β), it does significantly improve cardiopulmonary function in HIV-infected patients (23).

The importance of rehabilitation medicine is also evident from a 2019 literature review on HIV-infected children with impairments and disabilities. Unfortunately, we know that pediatric health systems in Sub-Saharan Africa are not integrated with rehabilitation in chronic diseases such as HIV while integration to pediatric Rehabilitation in a holistic approach would be important. This scoping review proposes a synthesis of existing evidence on rehabilitation intervention strategies for disability-related barriers in children living in Sub-Saharan Africa (24).

The incidence of diabetes mellitus (DM) in Africa is not only a health problem but also imposes a significant economic burden. Diabetic peripheral neuropathy is a common microvascular complication of DM that increases the potential for morbidity and disability due to ulceration and amputation. Based on the study analysis, the highest prevalence of diabetic peripheral neuropathy in patients with DM was reported in West Africa at 49.4%. The need for a rehabilitation medicine approach also has its importance here (25).

Despite concerns about underreporting of cerebral palsy (CP) in many African communities, the prevalence estimates reported here were generally higher than the estimated 2–2.5 of 1,000 in most studies conducted in the United States or Europe (26). It is likely that in Africa the prevalence of CP is high because of the level of perinatal complications such as birth asphyxia and neonatal infections. What is clear is that there is a lack of screening policy for disabilities among infants and pre-school children in Africa (27).

There is a paucity of studies in Africa, South-East Asia and the Eastern Mediterranean region on pulmonary diseases, with increasing prevalence of chronic obstructive pulmonary disease, both globally and regionally (28).

In the same way patients with idiopathic pulmonary fibrosis (IPF) generally experience poor quality of life. A study reports that these patients are poorly referred to palliative care even in developed countries, while in developing countries no data are available on the use of palliative care or the burden of health care management. Therefore, more awareness and research on the palliative care needs of patients with IPF is recommended, particularly in resource-limited settings such as South Africa (29).

About the burden of stroke in Africa, the results of a review suggest that is high and still rising. The incidence of stroke in Africa is becoming a public health challenge; unfortunately scarcity of data has limited research and consequently also the response to the exact public health burden.

In 2019, a total of 1.89 million stroke survivors were estimated among people aged 15 years or older in Africa. There is a need for extensive research on both stroke and other vascular risk factors to institute appropriate policy, and effective preventive and management measures (30).

Regarding the rheumatologic diseases, a systematic review identified the paucity of latest prevalence data on arthritis in Africa (31).

After this excursus of the most important diseases that afflict the African continent, this systematic review of the empirical literature, from 2016, emphasizes the importance of exploring the sustainability of health interventions in Sub-Saharan Africa.

From the analysis of these studies, we can define the importance of rehabilitation and the need for more studies in this area (32).

For the application of proper rehabilitation in the field of PRM, a study emphasizes the need to understand the current learning styles of physiotherapy students and if necessary also change the teaching styles in order to provide high quality education. Currently, physiotherapy students have specific learning styles of active participation supported by practical internship activities and theoretical concepts. Further research would be fundamental to define and standardize learning styles in physiotherapy courses (33).

The results of a study published in 2009 offer the first global portrait of the dynamics of demand and supply of human resources for rehabilitation: the lowest supply of rehabilitation health professionals was found among low- and middle-income countries, many located in Sub-Saharan Africa, where the burden of cause-related diseases requiring rehabilitation professional skills tends to be greatest. Worldwide, people with disabilities have many unmet health and rehabilitation needs but continue to face significant barriers in accessing mainstream health services, and consequently have poorer health outcomes. Currently, a double burden is found in low- and middle-income countries. Unfortunately, human resources for rehabilitation are often a neglected component of health services (34).

## Discussion

In light of what has been examined so far, we discuss the current situation of disability and PRM in the Cameroon healthcare system and their possible perspectives.

### Where Is Disability in the Cameroon Healthcare System?

Healthcare system promotes equity in people. In addition, WHO reported that people with disabilities are also entitled to attain the best possible quality of care without discrimination. In the same vein, Cameroon has signed and ratified numerous national and international conventions on disability with the aim to attain a number of privileges for disabled persons, which have been recently characterized by Foti et al. (17). It is including medical, material, financial and psychosocial assistance and other forms of assistance depending on the degree of disability. However, in practice Cameroon faces to several challenges of poor health system like other countries in Africa (17). Relative lack of a value-based reimbursement system for care act in general population, dearth of specialized medical structures and inadequate health care for disabled persons in Cameroon are the most noted. To address these challenges of poor health outcomes, the Cameroon healthcare system has established the Affordable Care Act, which aims to lower costs and improve quality. Also, to answer this situation, training physicians in PRM is an opportunity.

### PRM Within the Cameroon Medical System

Rehabilitation aims to optimize functional ability, enhance quality of life and reduce disability in people with impaired health conditions through interventions. According to the WHO, countries with the lowest levels of health (and education) fail to sustain real growth and development (35, 36). Are physicians in Cameroon prepared to adequately prescribe exercise-based rehabilitation?

The current Cameroon medical system woefully underprepares clinicians to efficiently prescribe exercise-based rehabilitation. In addition, the majority of fellowships offer no training in exercise prescriptions. In Cameroon, the PRM curricula are not available in the existing medical schools. However, general physiotherapy and/or rehabilitation, speech therapy, occupational therapy, orthoprosthesis and psychomotricity programs are offered by some universities and institutions in Cameroon as elective teaching modules. Therefore, PRM as a medical specialty is not well-known.

### Education and Research in the Field of PRM in Cameroon: Call to Action

Curriculum is an initial step for the development of any field and guide health research. In Cameroon, research in the field of rehabilitation also suffers. Thus, we propose some following points for a call to action to implement and disseminate this impactful discipline in Cameroon:

(1) Develop a 4-year higher specialty training program in PRM;
(2) Gain recognition of the new specialty in Cameroon by the Ministry of Higher Education;
(3) Establish a system such as training and research in rehabilitation to ensure continuity competence of physicians practicing PRM in Cameroon, according to recommendations and standards provided by international PRM boards: “To acquire the wide field of competence needed, specialists in Physical and Rehabilitation Medicine have to undergo a well-organized and appropriately structured training of adequate duration. In fact they are required to develop not only medical knowledge, but also competence in patient care, specific procedural skills, and attitudes toward interpersonal relationship and communication, profound understanding of the main principles of medical ethics and public health, ability to apply policies of care and prevention for disabled people, capacity to master strategies for reintegration of disabled people into society, apply principles of quality assurance and promote a practice-based continuous professional development” (37, 38).

## Conclusion

We can confirm that high-quality rehabilitation care is essential within health systems, especially for low- and middle-income countries. The importance of adapting staff training both at university and post-graduate level is a fundamental element to ensure the highest standard of rehabilitation care.

The review carried out on the literature relating to the main diseases that cause disabilities in Africa with particular regard to Cameroon highlighted shortcomings in the health systems both at a social and welfare level; for this reason, the aim is to define training courses and strategies that can guarantee the best level training to provide better intervention systems for professionals in PRM. This is why the international bodies of PRM highlight the need to develop training that allows education to be aligned with the priorities of the health system.

As regards Cameroon, a fundamental aspect would be to recognize the specialization in PRM by the Ministry of Higher Education, developing a 3 or 4-year educational program and improving scientific research in this field.

## Author Contributions

All authors listed have made a substantial, direct, and intellectual contribution to the work and approved it for publication.

## Funding

Publication of article was funded by Chair of Physical Medicine and Rehabilitation, Clinical Sciences and Translational Medicine Department, Tor Vergata University, Rome, Italy.

## Conflict of Interest

The authors declare that the research was conducted in the absence of any commercial or financial relationships that could be construed as a potential conflict of interest.

## Publisher's Note

All claims expressed in this article are solely those of the authors and do not necessarily represent those of their affiliated organizations, or those of the publisher, the editors and the reviewers. Any product that may be evaluated in this article, or claim that may be made by its manufacturer, is not guaranteed or endorsed by the publisher.
